# Topographic Changes in SARS Coronavirus–infected Cells at Late Stages of Infection

**DOI:** 10.3201/eid1011.040195

**Published:** 2004-11

**Authors:** M.L. Ng, J.W.M. Lee, M.L.N. Leong, A.-E. Ling, H.-C. Tan, E.E. Ooi

**Affiliations:** * National University of Singapore, Singapore;; † Singapore General Hospital, Singapore;; ‡ National Environment Agency, Singapore

**Keywords:** SARS coronavirus replication, atomic force microscopy, scanning electron microscopy, research

## Abstract

Scanning electron and atomic force microscopy was used for the first time to view the maturation of the severe acute respiratory syndrome–associated coronavirus at the cell surface. The surface form of the cells at advanced infection displayed prolific pseudopodia that, in addition to the rest of the plasma membrane, were also active sites of virus release. High magnification of the maturing virus particles showed a rosette appearance with short knoblike spikes under both the scanning electron and atomic force microscopes. The final expulsion step of the maturing virus particles seemed to result in some disruptions to the plasma membrane. The cytoskeletal network along the edge of the infected cells was enhanced and could be involved in transporting and expelling the progeny virus particles. Thickening of the actin filaments at the cell edge provided the bending force to extrude the virus particles.

## Abstract 2

Scanning electron and atomic force microscopy was used for the first time to view the maturation of SARS-CoV at the cell surface.

A new human coronavirus was identified during the recent outbreak of severe acute respiratory syndrome (SARS) (*1**–**4*). The outbreak started during November 2002 in southern China and then spread to Hong Kong, Vietnam, Canada, and Singapore in early 2003. Sequence analyses of various isolates have indicated that the virus is genetically distinct from all known coronaviruses (*5**–**7*). Phylogenetic analysis suggests that the SARS-associated coronavirus (SARS-CoV) does not fit in the three currently known groups of coronaviruses (*1**,**5**,**6**,**8*), which suggests that this is a new virus, not a result of mutation or recombination of known coronaviruses.

Coronavirus infections are common in both domestic animals and humans (*9*). However, the known human coronaviruses often cause coldlike symptoms, whereas recent infections caused by SARS-CoV do not. The rate of death for SARS infections is 7%–10%, depending on the age of the patients (*2*).

SARS-CoV grows well in Vero E6 cells (*1**,**2**,**10*) and enters cells by direct fusion of the virus envelope with the plasma membrane (*11*). The fusion process involving the S glycoprotein is pH independent (*12*). Once internalized, the virus core uncoats, revealing flattened, disc-shaped, and electron-dense nucleocapsids described as "doughnut-shaped" (*10**,**11*). The uncoated nucleocapsids are found within large, smooth, double-membrane vacuoles together with membrane whorls (*11*). These membrane whorls are postulated to be replication complexes for the virus since they appear very early (within 30 min) after infection. Other reports have described double-membrane vesicles as sites of replication for coronavirus (Linder strain) (*13*), mouse hepatitis virus (*14*), and SARS virus (*15*). The latent period observed was 5–6 h postinfection (*10*). However, a short latent period is common among coronaviruses (*16*).

Coronavirus infections can be cytocidal for the cells; or, in some cases, persistent infection can result (*17*). The outcome of the infection is dependent on the virus strains and cell types. Unlike infection with the hepatitis C virus-229E, wherein virus production can continue for weeks without any expression of cytopathic effects (*18**,**19*), infection with SARS-CoV produces copious progeny virus particles within the first 12 h (*10*). The site of assembly of SARS-CoV was at the Golgi complexes, similar to previous reports for other coronaviruses (*20**–**22*). After assembly, the virus progeny particles are transported in vesicles to the cell periphery for release.

The aim of this study was to use scanning electron and atomic force microscopes to investigate changes in the surface topography of SARS-CoV–infected cells at late infection. The results can assist in further understanding how SARS-CoV interacts with infected cells at late infection. Thus far, replication studies on SARS-CoV were performed with transmission electron microscopy, which showed detailed intracellular changes during replication in two dimensions. Both scanning electron and atomic force microscopy can provide holistic and three-dimensional views as infection progresses.

## Materials and Methods

### Cells and Virus

SARS-CoV (2003VA2774) used for this study was isolated from a SARS patient in Singapore by the Department of Pathology, Singapore General Hospital. The virus was grown in Vero E6 cells (ATCC: C1008) in the Environmental Health Institute, National Environmental Agency, Singapore. Infection of the cells grown on coverslips and subsequent fixation (5% glutaraldehyde) of the infected cells at appropriate times were performed at that institute. The microscopy work on the fixed infected cells was performed at the Electron Microscopy Unit, National University of Singapore.

### Scanning Electron Microscopy

Vero cells were grown to 70% confluency on sterile glass coverslips in 24-well tissue culture plates before infection with 100 µL of SARS-CoV for 1 h (multiplicity of infection = 10). Maintenance media supplemented with 2% fetal calf serum was added to the wells, and the infected cells were incubated in 37°C incubator with 5% carbon dioxide.

At an appropriate time after infection, the infected cells on the coverslips were fixed with 5% glutaraldehyde overnight. The coverslips were washed with phosphate-buffered saline before being postfixed in 1% osmium tetroxide for 1 h. The coverslips were then washed with distilled water and dehydrated through a series of increasing concentration (25%–100%) of ethanol. Cells on the coverslips were further subjected to critical point drying for 1.5 h and left in a 37°C oven overnight. Subsequently, the cells on the coverslips were sputter-coated with gold (thickness of 10 nm) and viewed under the XL30 Field Emission Gun scanning electron microscope (FEI Company, Enidhoven, the Netherlands) at 10 kV.

### Atomic Force Microscopy

Infected cells were processed similarly. Normally, samples for the atomic force microscopy should be subjected to minimal processing so that the samples are close to their natural condition. However, in view of the pathogenicity of SARS-CoV, only fixed and gold-coated samples were used for this study. The NanoScope IV MultiMode atomic force microscope was used (Veeco Instruments, Woodbury, NY). Force modulation etched silicon probes were used for imaging (dry TappingMode [Vecco]) infected cells. Hard tapping using appropriate amplitude setpoints was performed with some samples to show subsurface structures.

### Negative Staining

Purified virus fixed in 2.5% glutaraldehyde was put onto a formvar carbon-coated grid and allowed to adsorb for a few minutes before being stained with 1% phosphotungstenic acid for 1 min. The excess fluid was blotted and the grid left to dry before viewing under CM120 BioTwin TEM (FEI Company, Enidhoven, the Netherlands).

## Results

**Figure 1:**
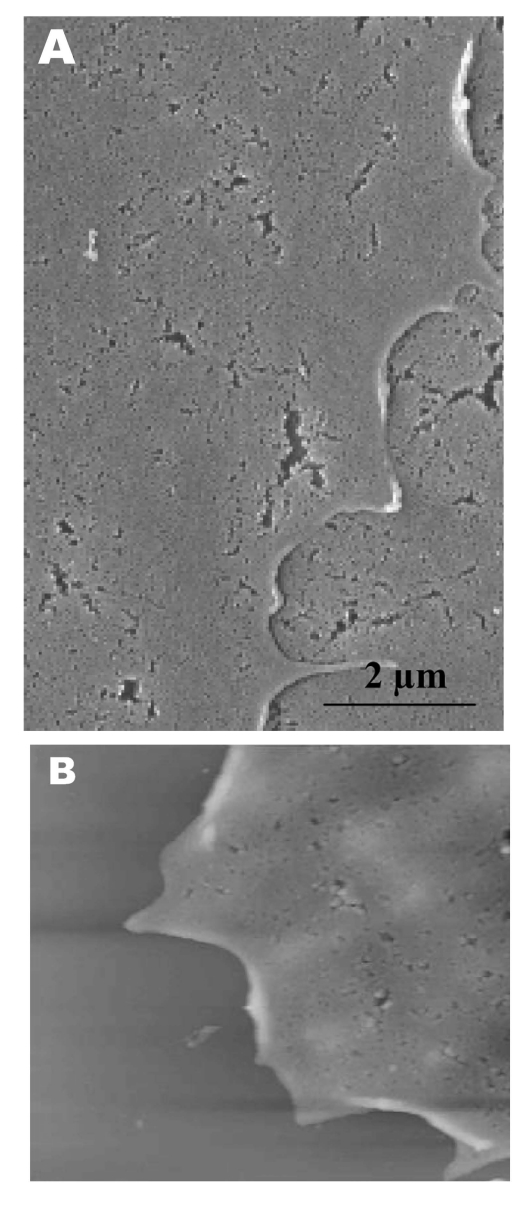
Scanning electron (A) and atomic force (B) microscopy images of uninfected Vero cells. A) Under the scanning electron microscope, uninfected cells look relatively flat with minimal surface morphology. No pronounced pseudopodia are visible on the cell edge or surfaces. B) Atomic force microscopy confirms the form and structure seen in panel A. Cell surface is uniformly flat.

Both scanning electron and atomic force microscopy showed that the uninfected Vero cells were flat and without prominent form and surface (Figure 1). Pseudopodia, where present, were not extensive (Figure 1A and B).

**Figure 2:**
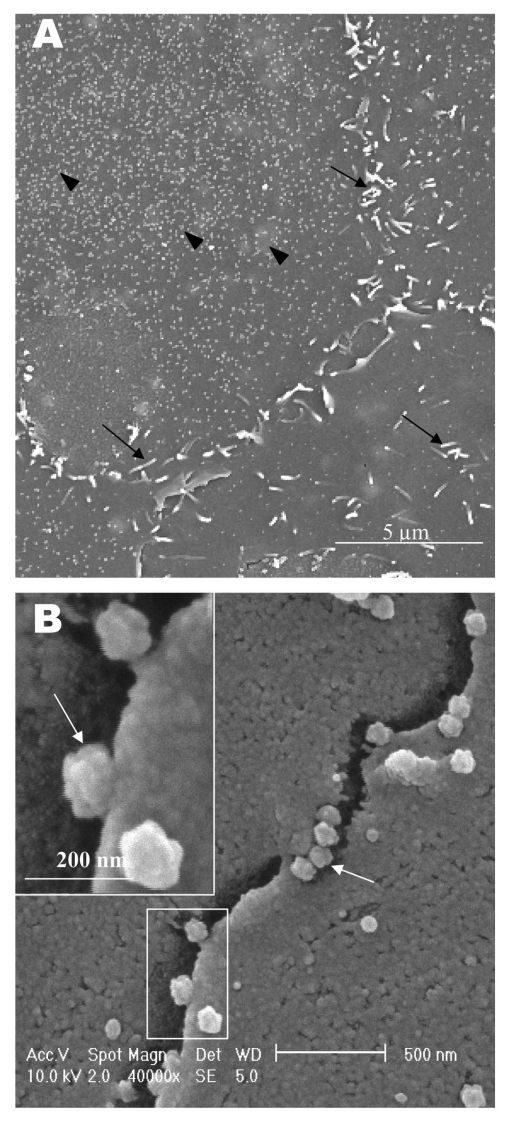
Scanning electron microscopy of Vero E6 cells infected with severe acute respiratory syndrome–associated coronavirus at 15 h after infection. A) One pronounced surface morphologic change is the proliferation of psuedopodia at the cell periphery (arrows). Some pseudopodia are also developing on the cell surface. Some cells appear to have large amount of extracellular virus on the cell surface (arrowhead), whereas neighboring cells seem deprived of any extracelluar virus particles. B) Virus particles are protruding from the edge of cells (arrows). Inset shows the boxed area at higher magnification. Virus particles appear knobby and rosettelike.

In the transmission electron microscopy studies (*10**,**11**,**15*), SARS-CoV replicated very rapidly and produced large amounts of virus after 6 h of infection. The scanning electron microscope confirmed that, for some infected cells (15 h postinfection), a large quantity of extracellular virus was present (Figure 2A, arrowheads) on the whole cell surface. However, very few virus particles were on the neighboring cell (top right), indicating a nonsynchronous infection. The scanning electron microscopy images showed a holistic view of SARS-CoV–infected cells compared to ultrathin sections in transmission electron microscopy. Another virus-induced change clearly demonstrated by using the scanning electron microscope was the proliferation of pseudopodia on the infected cells and in particular, at the edge of these cells (Figure 2A, arrows compared to Figure 1).

At higher magnification, progeny virus particles protruded at the cell periphery (Figure 2B, arrow). In the inset (boxed area), a virus particle was seen in the process of extrusion (arrow) after the fusion of the transport vesicle and the plasma membrane. The knoblike spikes surrounding the coronavirus were clearly visible. SARS-CoV spikes appeared short and stubby (16–17 nm) when compared to those of other coronaviruses (20 nm). This feature gave the virus a rosettelike appearance when viewed under the scanning electron microscope (arrowheads indicate extruded virus particles). The average size of the extracellular virus particles was 100–130 nm. The gold sputter coating can also increase in the diameters of the virus particles.

**Figure 3:**
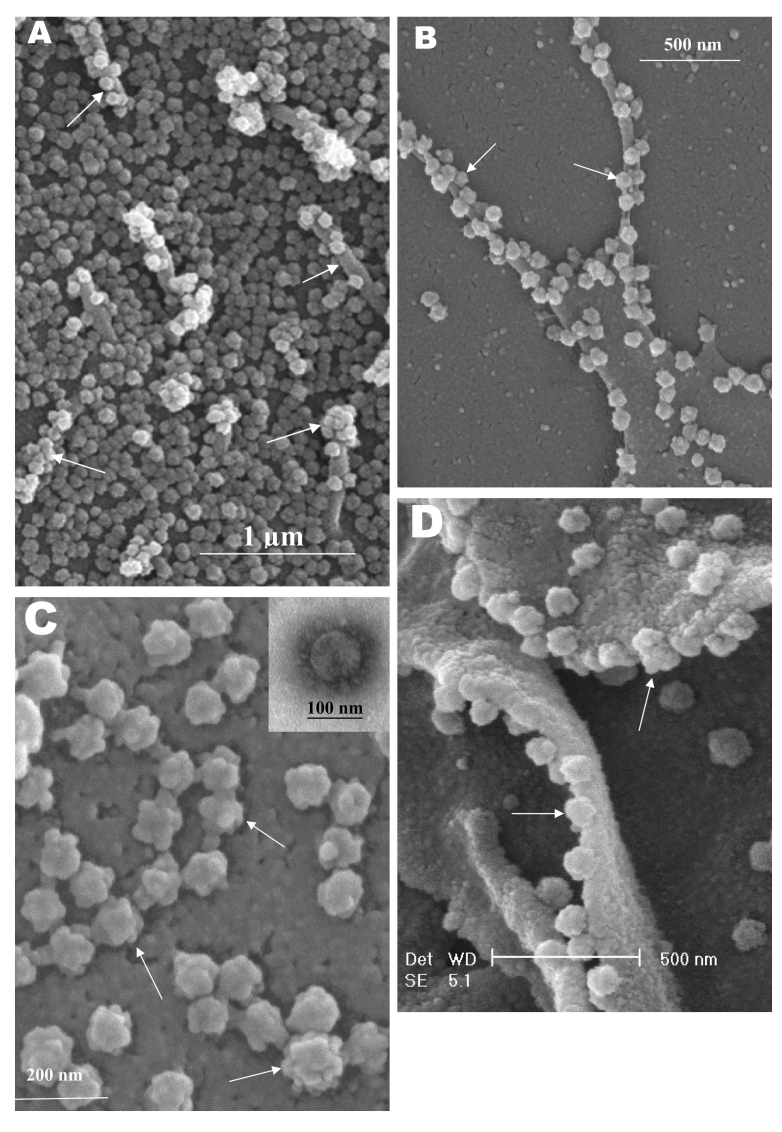
Scanning electron microscopy of Vero E6 cells infected with severe acute respiratory syndrome–associated coronavirus at 24 h after infection. A) Cell surface is covered with extracellular progeny virus particles, and progeny virus are being extruded from or attached to numerous pseudopodia on infected cell surface (arrows). B) A higher magnification micrograph of the virus-clustered pseudopodia (arrows). C) Rosettelike appearance of the matured virus particles (arrows). The scanning electron microscopy image complements the form and structure of the virus seen with negative staining (inset) under transmission electron microscopy. Short and stubby spikes are visible on the virus surface. D) Arrows indicate virus particles being exported from the surfaces of the filopodia.

From 15 to 24 h after infection, the virus was exported prolifically at the pseudopodia and cell surfaces (Figure 3A, B, and C, arrows). The surface imaging clearly showed the profuse presence of extracellular virus (arrows). High magnification scanning electron microscopy images of the SARS-CoV form and structure (Figure 3C, arrows) appeared to correlate well with those images that used negative staining and TEM (Figure 3C, inset). The knoblike spikes were short and stubby in the negative staining image as well. Figure 3D shows virus particles were also exported out from the surface of the pseudopodia (arrows).

**Figure 4:**
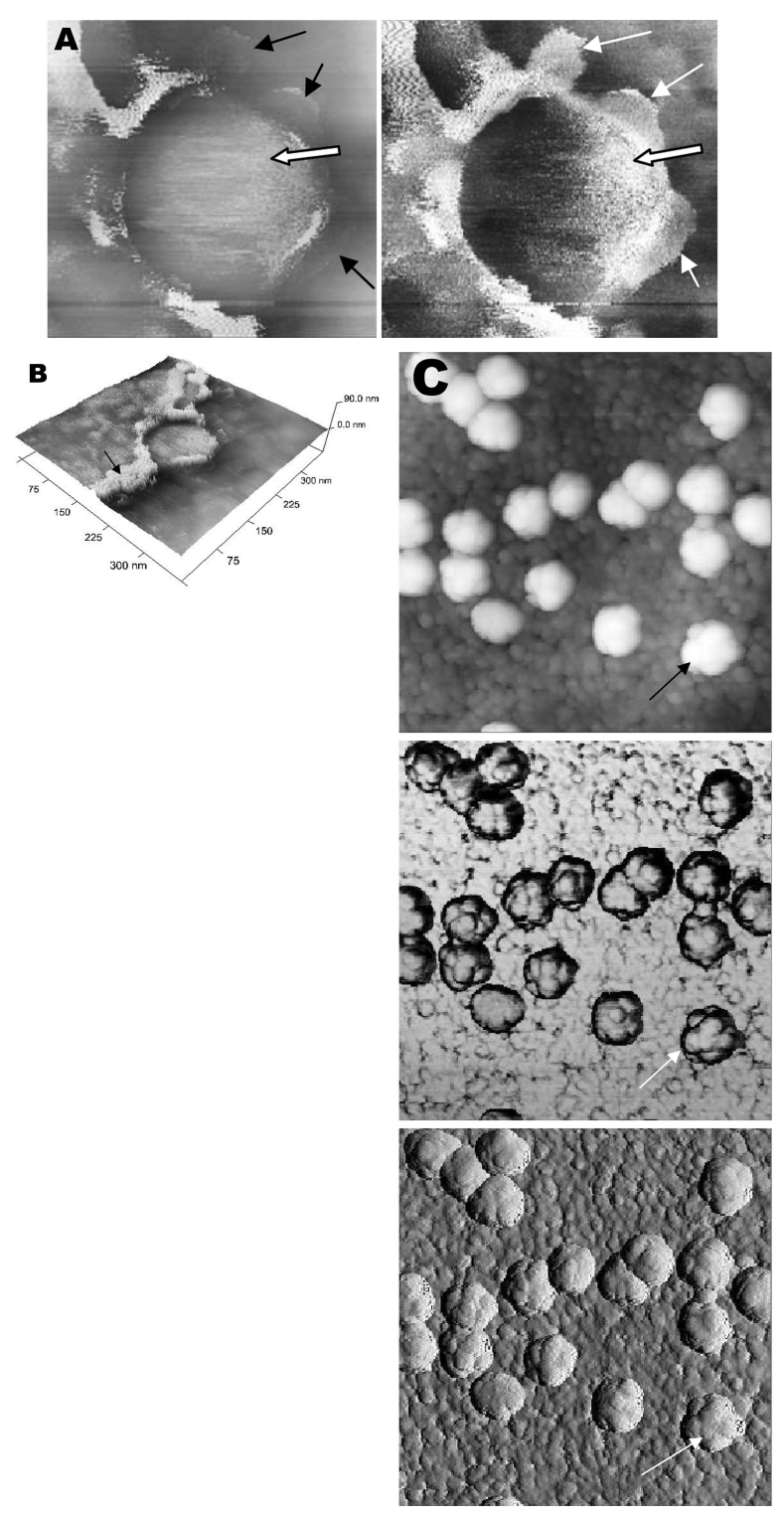
Atomic force microscopy of Vero cells infected with severe acute respiratory syndrome–associated coronavirus at 15 h after infection. A) At much higher resolution imaging of the edge of a cell, a virus particle (thick arrow) in the process of extruding from the cell plasma membrane (PM) after fusion of the transport vesicle with the cell membrane. PM shows some loss of integrity (thin arrows) during this exit process. B) A three-dimensional reconstruction of the extruding virus particle from panel B. Arrow indicates the thickened cell edge. C) Arrow indicates the knoblike structures on the virus particles.

A virus particle in the process of extrusion at the cell plasma membrane was captured with the atomic force microscope at 15 hours after infection. Although the proposed mechanism for export of the virus to the extracellular space is through fusion of the transport vesicle membrane at the cell surface, this process seemed to result in localized breaching at the plasma membrane, where the virus extrusion occurred (Figure 4A, thin arrows). Although fixed and gold-coated samples were used in this study, the atomic force microscope delivered high-resolution images. Unfortunately, the knoblike spikes for this virus were not well illustrated in Figure 4A. A three-dimensional reconstruction (Figure 4B) shows that the virus particle was extruding from a much-thickened cell periphery (arrow). The knoblike structures on the virus surface were further confirmed by atomic force microscopy (Figure 4C).

**Figure 5:**
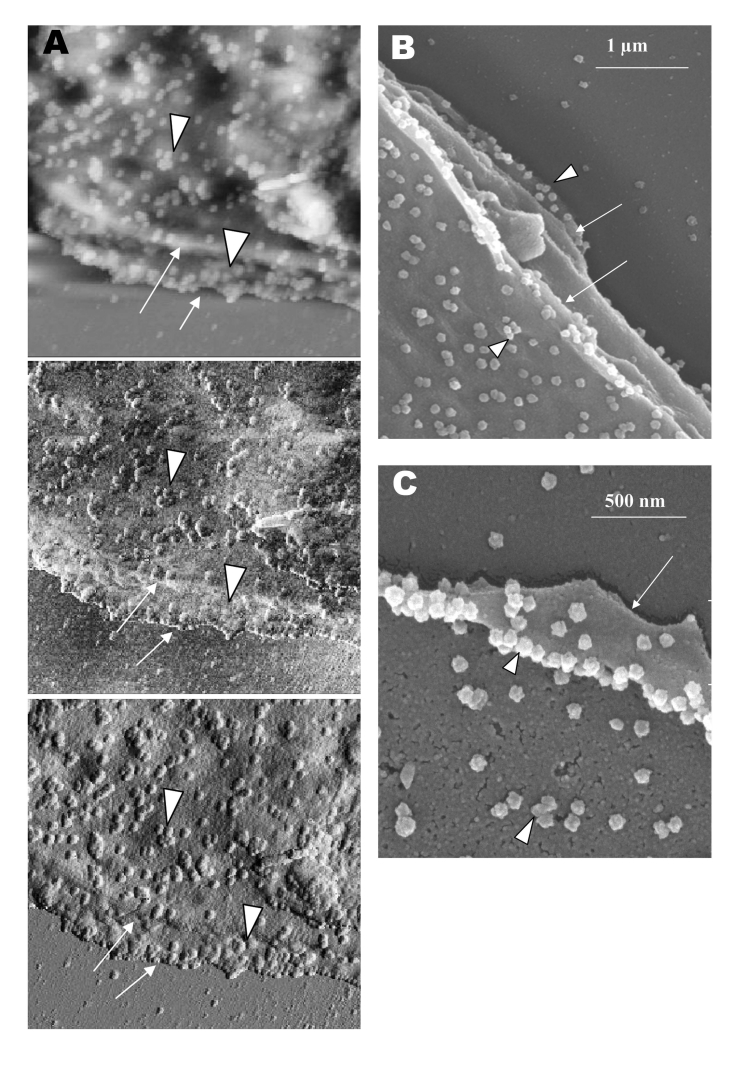
Vero cells infected with severe acute respiratory syndrome–associated coronavirus. A) An atomic force microscopy image of the thickened, layered appearance of the edge of the cells (arrows), where active virus extrusion occurs. Arrowheads indicate the virus particles. B) The layered cell edge (arrows) seen by scanning electron microscopy. Virus particles extrude from the layered surfaces. Arrowhead indicates virus particles. C) Virus particles (arrowheads) extrude from the layered surfaces (arrows).

The thickened edges of the infected cells were ruffled and appeared to comprise layers of folded membranes (Figure 5A, B, and C). The layered/folded effects at the edge of cells were pronounced in the height image under the atomic force microscopy and scanning electron micrographs. The arrowheads show the virus particles.

**Figure 6:**
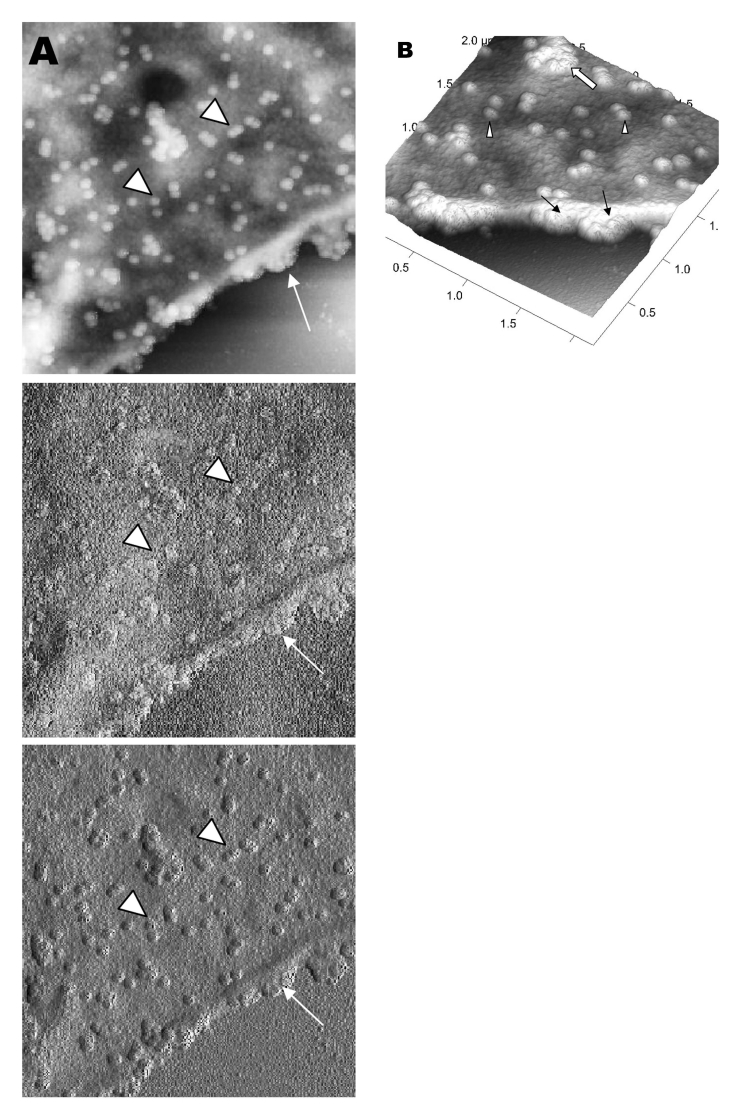
Atomic force microscopy of Vero cells infected with severe acute respiratory syndrome–associated coronavirus. A) High activity of virus extrusion at the thickened edge of the infected cells (arrow). Arrowheads indicate virus particles. B) A three-dimensional reconstruction of the image in panel A shows the puffy edge of infected cells. Many intracellular viruses are visible just under the plasma membrane (arrows). Extruded virus particles are present on other areas of the cell surface (arrowheads). Thick white arrow shows a large clump of virus particles just beneath the plasma membrane.

Virus particles (arrowheads) could still be exported out of the puffy edge (Figure 6A, arrows). A three-dimensional reconstruction (Figure 6B) of the height image in Figure 6A shows puffy fronts of the cell edge (arrows) with many virus particles just underneath the surface awaiting extrusion. The large number of progeny virus particles at the cell edge may have resulted in this thickened appearance. Virus particles (arrowheads) were present on other parts of the cell surface as well. Thick white arrow shows a clump of virus particles just underneath the plasma membrane.

**Figure 7:**
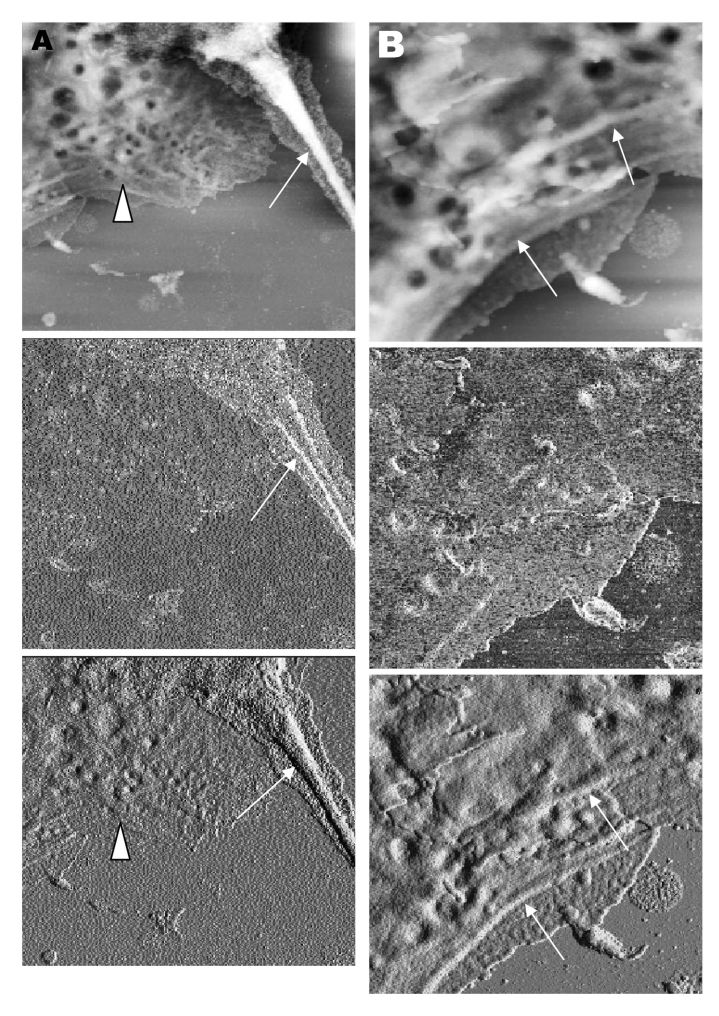
Vero cells infected with severe acute respiratory syndrome–associated coronavirus at 15 h after infection. A and B) When the hard tapping mode of the atomic force microscope is used, thickened cell cytoskeletal filaments are found below the subcellular surface of the cells. A) Enhanced cytoskeletal network of the cytoplasmic region (arrowhead). Arrow shows the much-thickened filaments within the pseudopodia of the cell. B) At high resolution, the arrows show the thickened cytoskeletal filaments along the cell periphery. The height and amplitude images clearly show the cytoskeletal filaments parallel to the cell edge.

Closer examination of the virus-induced changes at the subcellular surfaces of the infected cells, by using the hard tapping mode under the atomic force microscope, showed the involvement of the cell cytoskeleton at late infection. In Figure 7A, gross thickening of the cell skeletal filaments was seen in the cytoplasm (arrowhead) and pseudopodia (arrows). At higher resolution, thickening of the filaments at the edge of cells was obvious (Figure 7B, arrows). These filaments, which ran parallel to the cell edge, could be the enhanced actin filaments, and together with the accumulated progeny virus particles, could have caused the bulky, puffy-cell periphery.

## Discussion

By using transmission electron microscopy, recent studies (*10**,**11*) showed the entry events and prolific growth of SARS-CoV in Vero E6 cells. SARS-CoV enters the cell by direct fusion and has a latent period of only 6 h. High numbers of progeny virus particles assemble in the swollen Golgi sacs before export to the external surface.

Transmission electron microscopy of ultrathin sections gave good intracellular information but was not able to give a gross morphologic landscape of the infected cells. Surface topographic changes induced by SARS-CoV at maturation and late stages of infections were the focus of this study. The virus-induced modifications at the cell surface or subcellular surface could relate to the eventual destruction of the infected cells as well as shed light on the extrusion mechanism of the progeny virus particles from the cell surface.

Scanning electron microscopy, an established technique, gives a three-dimensional overview of the virus and the infected cell surfaces. Another high-resolution device used in this study is the atomic force microscope. It is also gaining popularity in areas of life science research (*23**–**29*). Most of these studies were on purified macromolecules. However, the atomic force microscope has also become a virologic standard in recent years (*30**–**33*). A recent study on HIV and HIV-infected lymphocytes (*34*) demonstrated the strength of this technique for virology.

The application of these two selected techniques to study the late SARS virus–induced changes in Vero cells was rewarding. The SARS-CoV knobby/rosettelike structures were seen in a three-dimensional form under the scanning electron and atomic microscopy (Figure 2B, C, Figure 4C). The spikes seemed shorter (16–17 nm) than those of other coronaviruses. At this stage, it is speculative if this could be due to the lack of the hemagglutinin-esterase protein (*8**,**35*) in the spike glycoprotein of this virus. Further structural and functional studies should be performed to investigate this aspect and its relation to virus virulence.

The scanning electron microscopy studies showed prolific SARS-CoV on infected cell surface 15 hours after infection. Unlike ultrathin sectioning in transmission electron microscopy, the scanning techniques allow cell and virus surfaces to be viewed without invasive manipulation. In addition to the large amount of extracellular virus particles on most cells, proliferation of the pseudopodia in the infected cells was pronounced (Figure 2A compared to Figure 1). These pseudopodia increase the surface area of the cells as active maturation sites of virus (Figure 3A and B).

Although the scanning electron microscope was able to show virus particles in the process of extruding (Figure 2B, Figure 3A and B) from the cells, the image derived with the atomic force microscope was superior in resolution. A virus particle was seen pushing out of the cell plasma membrane (Figure 4A), which resulted in localized loss of membrane integrity at the site. Since prolific extrusion of the progeny virus particles occurred at this late stage of infection, the frequent loss of plasma membrane integrity could compromise the physiologic status of the infected cells and lead to cell death.

Fifteen hours after infection, ruffled, puffy peripheries were visible in infected cells (Figure 4B, Figure 5, and Figure 6) and not seen in uninfected cells (Figure 1). This feature was not obvious under the transmission electron microscopy (*11*). Subcellular imaging of the thickened edge of the cells showed numerous progeny virus particles awaiting extrusion (Figure 6B, arrows). The actin filaments that were parallel to the cell edge appeared to have thickened (Figure 7A and B compared to Figure 1A and B). The enhanced presence of the actin filaments could assist in providing the bending force to expel the progeny virus particles to the exterior. Bohn and colleagues (*36*) suggested that the forces resulting from the vectorial growth of the actin filaments contributed to membrane bending at the site of virus maturation. Actin filaments have also been reported to be directly involved in the budding of both enveloped DNA and RNA viruses (*37**–**40*).

In summary, the cellular cytoskeleton network is involved in the SARS-CoV maturation and possibly replication process. The constant loss of membrane integrity attributable to the prolific progeny virus extrusion resulted in disintegration of infected cells.
